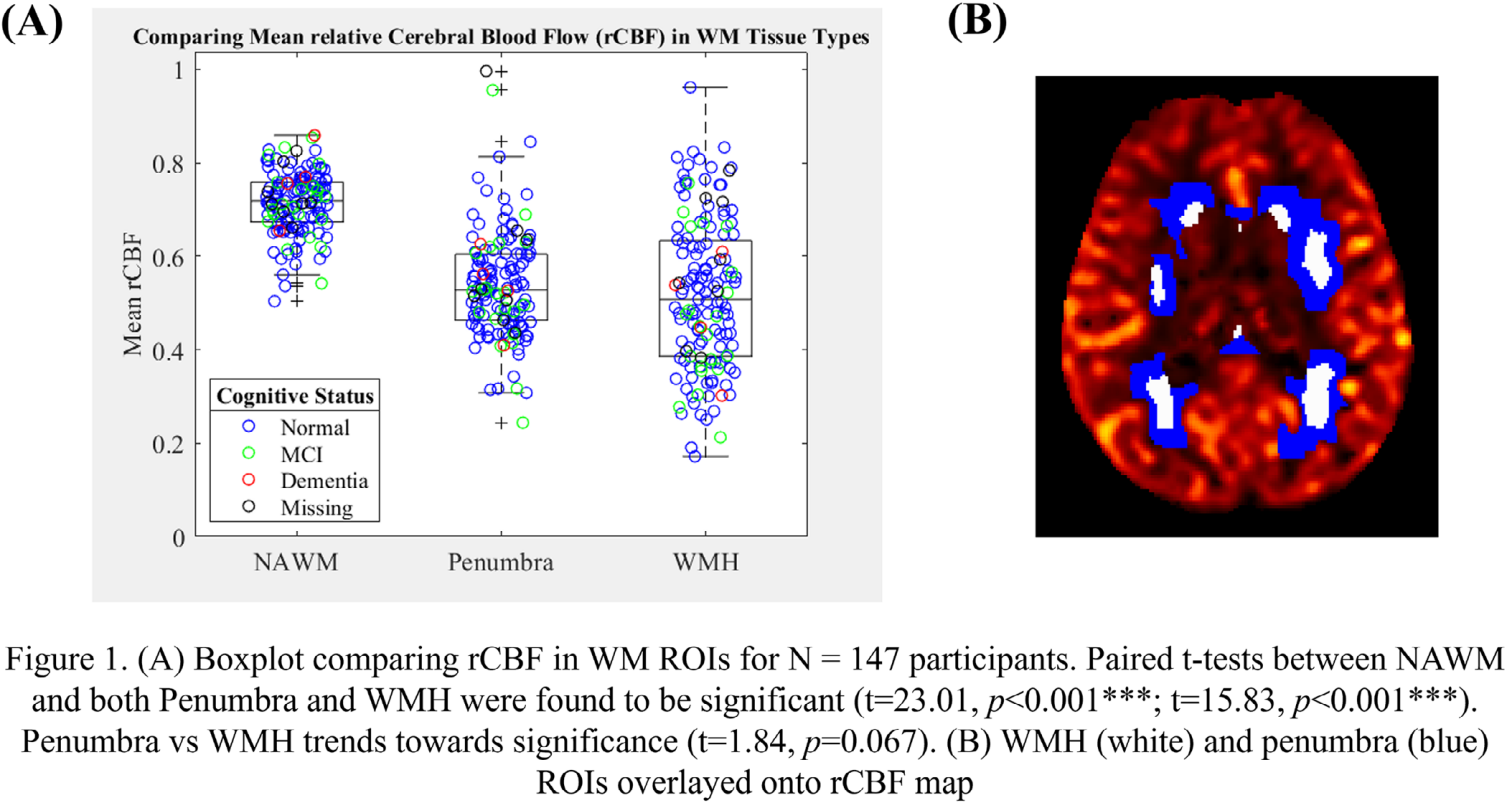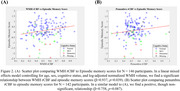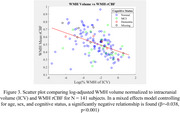# Associations between white matter hyperintensities (WMH), relative cerebral blood flow (rCBF), and cognition using multi‐delay ASL

**DOI:** 10.1002/alz70861_108666

**Published:** 2025-12-23

**Authors:** Tyler Statema, Youngkyoo Jung, Pauline Maillard, Sarah Tomaszewski Farias, Charles DeCarli, Audrey P. Fan

**Affiliations:** ^1^ University of California, Davis, CA USA

## Abstract

**Background:**

White matter hyperintensity (WMH) burden has been associated with cognitive impairment, increased risk of stroke, and increased risk of dementia (DeCarli, 1995; Wardlaw, 2015). Additionally, lower cerebral blood flow (CBF) has been associated with cognitive impairment and cerebrovascular damage (Soman, 2021). We used multi‐delay arterial spin labeling (ASL) MRI to measure regional perfusion in an older, diverse, non‐stroke impacted population and investigated CBF associations with WMHs and cognition.

**Methods:**

MRI at 3‐Tesla was performed for N=153 participants, CN=117, MCI=23, 5 with dementia, and 8 unknown cognitive status. There were 101 females and 52 males, a mean age of 74.1+/‐6.4 years. T1‐weighted and T2‐FLAIR scans were segmented to obtain grey matter, white matter (WM), and WMH masks. WMH clusters were dilated by 8mm to create penumbra regions (Maillard, 2011). Individual CBF maps were produced through BASIL, fitting a kinetic model using 5 post‐label delays. These were then normalized to obtain relative CBF (rCBF) maps, the mean of which was calculated for WMH, penumbra, and normal‐appearing WM (NAWM) ROIs in T1 space. Cognitive measures were assessed within 6 months of the MRI scans following SENAS methods (Mungas, 2004).

**Results:**

We found a significant relationship between rCBF and WM tissue type (β=‐0.089, *p* <0.001) while controlling for age (Figure 1). We also find a positive relationship between WMH rCBF and episodic memory score while controlling for age, sex and cognitive status (β=0.937, *p* =0.039) (Figure 2A), in addition to a nonsignificant trend when examining penumbra rCBF (β=0.758, *p* =0.087) (Figure 2B). Finally, we found a significant negative relationship between WMH rCBF and log‐adjusted normalized WMH volume while controlling for sex, age, and cognitive status (β=‐0.038, *p* <0.001) (Figure 3).

**Conclusions:**

The finding that rCBF is lower in WMH and surrounding penumbra regions is in line with previous research (Promjunyakul, 2015) and may indicate that hypoperfusion and vascular pathophysiology underlie WMH development. The finding of a negative correlation between WMH volume and WMH rCBF may indicate a more severe impact of hyperintensities as WMH pathophysiology progresses. The positive association between WMH rCBF and episodic memory further supports the need to study hyperintensities in the context of both aging and cognition.